# Cancer in The Gambia: 1988–97

**DOI:** 10.1054/bjoc.2001.1730

**Published:** 2001-05

**Authors:** E Bah, D M Parkin, A J Hall, A D Jack, H Whittle

**Affiliations:** International Agency for Research on Cancer, c/o The Gambia Hepatitis Intervention Study, MRC Laboratories, Fajara, PO. Box 273, Banjul, The Gambia; International Agency for Research on Cancer, Lyon, France; London School of Hygiene and Tropical Medicine, London, UK; World Health Organization Representative's Office, Gaborone, Botswana; Medical Research Council Laboratories, Fajara, PO Box 273, Banjul, The Gambia

**Keywords:** incidence, cancer, registration, hepatitis B, immunization

## Abstract

We describe the incidence of cancer in The Gambia over a 10-year period using data collected through the Gambian National Cancer Registry. Major problems involved with cancer registration in a developing country, specifically in Africa are discussed. The data accumulated show a low overall rate of cancer incidence compared to more developed parts of the world. The overall age standardized incidence rates (ASR) were 61.0 and 55.7 per 100 000 for males and females, respectively. In males, liver cancer was most frequent, comprising 58% of cases (ASR 35.7) followed by non-Hodgkin lymphoma, 5.4% (ASR 2.4), lung 4.0%, (ASR 2.8) and prostate 3.3% (ASR 2.5) cancers. The most frequent cancers in females were cervix uteri 34.0% (ASR 18.9), liver 19.4% (ASR 11.2), breast 9.2% (ASR 5.5) and ovary 3.2% (ASR 1.6). The data indicate that cancers of the liver and cervix are the most prevalent cancers, and are likely to be due to infectious agents. It is hoped that immunization of children under 1 year against hepatitis B will drastically reduce the incidence of liver cancer in The Gambia. © 2001 Cancer Research Campaign http://www.bjcancer.com


*British Journal of Cancer* (2001) **84**(9), 1207–1214
© 2001 Cancer Research Campaign
doi: 10.1054/ bjoc.2001.1730, available online at http://www.idealibrary.com on IDEAL®

### Cancer in The Gambia: 1988–97

**E Bah[1], DM Parkin[2], AJ Hall[3], AD Jack[4] and H Whittle[5]**

[1]International Agency for Research on Cancer, c/o The Gambia Hepatitis Intervention Study, MRC Laboratories, Fajara, PO. Box 273, Banjul, The Gambia; [2]International Agency for Research on Cancer, Lyon, France; [3]London School of Hygiene and Tropical Medicine, London, UK; [4]World Health Organization Representative's Office, Gaborone, Botswana; [5] Medical Research Council Laboratories, Fajara, PO Box 273, Banjul, The Gambia

**Summary** We describe the incidence of cancer in The Gambia over a 10-year period using data collected through the Gambian National Cancer Registry. Major problems involved with cancer registration in a developing country, specifically in Africa are discussed. The data accumulated show a low overall rate of cancer incidence compared to more developed parts of the world. The overall age standardized incidence rates (ASR) were 61.0 and 55.7 per 100 000 for males and females, respectively. In males, liver cancer was most frequent, comprising 58% of cases (ASR 35.7) followed by non-Hodgkin lymphoma, 5.4% (ASR 2.4), lung 4.0%, (ASR 2.8) and prostate 3.3% (ASR 2.5) cancers. The most frequent cancers in females were cervix uteri 34.0% (ASR 18.9), liver 19.4% (ASR 11.2), breast 9.2% (ASR 5.5) and ovary 3.2% (ASR 1.6). The data indicate that cancers of the liver and cervix are the most prevalent cancers, and are likely to be due to infectious agents. It is hoped that immunization of children under 1 year against hepatitis B will drastically reduce the incidence of liver cancer in The Gambia. © 2001 Cancer Research Campaign http://www.bjcancer.com

**Keywords**: incidence; cancer; registration; hepatitis B; immunization

The Gambia National Cancer Registry (GNCR) is the only population-based cancer registry in Africa with national coverage and the only one covering a substantial rural population. The registry was established as part of The Gambia Hepatitis Intervention Study (GHIS). GHIS was designed as a randomized control trial, with the main aim being to evaluate the effect of hepatitis B vaccination in infancy on subsequent risk of primary liver cancer (The Gambia Hepatitis Study Group, 1987). The GNCR was established to monitor the occurrence of hepatitis B-related liver disease, mainly primary liver cell carcinoma and cirrhosis of the liver, among the GHIS cohort. The registry provides a unique opportunity to describe the pattern of cancer occurrence and outcome in a predominantly rural population in sub-Saharan Africa. In this paper, we describe the incident cases of cancer recorded in the registry in the 10-year period 1988–1997.

### METHODS AND MATERIALS

The Gambia is a small country in West Africa (11 300 km$^2$) occupying a strip of land on both banks of the river Gambia (Figure 1). In 1999, the population was estimated to be 1.34 million. It is divided into 7 administrative districts (5 divisions and 2 municipalities), and more than 75% of the population are rural, engaged in peasant farming and stock rearing. Primary health care is delivered through village health posts, dispensaries and minor health centres. Qualified medical doctors at the major health centres and private 'non-profit' institutions provide secondary care. There are several private clinics, also, mainly in and around the capital city that deliver general medical care. There are 3 hospitals providing facilities for tertiary and/or specialist care in The Gambia, viz, Royal Victoria Hospital (RVH) in the capital city of Banjul, Bansang Hospital in the centre of the country and the Medical Research Council Laboratories of the United Kingdom (MRC), where the GNCR is located (see Figure 1). The RVH and Bansang hospitals are semi-autonomous government-owned institutions with services in surgery, dentistry, paediatrics, obstetrics & gynaecology, ophthalmology, pathology, radiology (X-ray only) and general medicine.

As part of efforts to improve diagnosis of cancer in the country for the final evaluation of the GHIS project, the International Agency for Research on Cancer (IARC) has assisted in the establishment of an in-country histopathology service. This service is at its infancy, located at the country's only histopathology laboratory based at the RVH.

Notification of cancer is voluntary in The Gambia. Doctors both in the public and private sector willing to collaborate are supplied with notification forms designed by the registry. In addition, data are collected actively by trained field staff from all health institutions that provide secondary or tertiary care. The sources of data include laboratory reports (mostly histopathology, haematology and biochemistry), patient case notes, ward admission and discharge, nursing report books, medical records ledgers and theatre record books.

Registration of death is incomplete in Gambia. A death certificate is only needed in order to obtain a permit for burial within the capital city, Banjul (with only 6% of the population), or for legal purposes. Copies of certificates mentioning cancer are obtained from the registration office. 'Death Certificate Only' cases are those for which no diagnostic information could be found from any other source.

Personal interviews with patients are also carried out by the trained field staff, so as to estimate age and determine usual place of residence and nationality. Accurate information on age, residence and nationality are sometimes difficult to obtain since most Gambians do not know their exact date of birth, and there is

*Received 3 November 2000*
*Revised 30 January 2001*
*Accepted 30 January 2001*

*Correspondence to:* E Bah

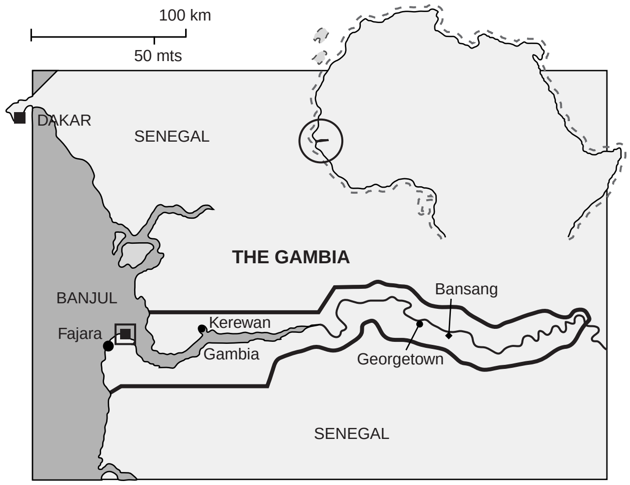

**Figure 1**

considerable migration across national or regional borders often in search of better health care. The Gambia's immigration service has issued national identity cards (ID) to all adult citizens (18 and above), the possession of which is mandatory by law. The ID contains information on age and usual place of residence. If a patient is not in possession of a valid ID card, the person is eligible for inclusion into the cancer register only if he or she has resided for 3 or more years in the country before first presentation of symptoms. This criterion effectively excludes non-residents who came to seek treatment in The Gambia.

The Registry is computerized and uses the CANREG-3 software developed by the International Agency for Research on Cancer (Cooke, 1998) which is used to search for duplicate registrations. The variables recorded for each patient include personal and demographic data, the site and histology of the tumour, and the source of information and basis of diagnosis. Tumour site and morphology are coded according to the ICD-O (second revision) (Percy et al, 1990).

The population at risk was derived from the national censuses of 1983 and 1993. Figure 2 shows the estimate for 1992 (assuming a constant growth rate between 1983 and 1993), the mid-point of the 10-year period considered here which was used to calculate incidence rates. Age standardization was carried out by the direct method using the world 'standard' population (Doll et al, 1966). Because of the large number of cases of unknown age (25% of the total), ASRS were calculated on the assumption that the age distribution of 'not known' cases was the same for each site as that of the known cases (Smith, 1992).

### RESULTS

Over the 10-year period (1988–1997), a total of 2957 malignant tumours were registered among residents of The Gambia. Over 60% of the cases were detected at the RVH and MRC. Overall 21% of the malignancies were verified through diagnostic pathology. Table 2 shows the percentage of histologically confirmed registrations for selected cancer sites. This ranges from more than 70% for superficial cancers for example skin melanomas or Kaposi's sarcoma, to 4% for deep sited tumours, notably liver cancers. Serum alpha-fetoprotein, and ultrasound examination were employed to confirm the clinical diagnosis of 75% of liver cancers. Less than 2% of cases were initially detected from death certificates without the availability of further information from other records to confirm the diagnosis.

Tables 1a and 1b show the numbers of cases by age group, together with the all age relative frequencies (%) and crude and age-standardized rate (ASR) for males and females respectively.

Among males, liver cancer was most frequent, comprising 58% of cases (ASR 35.7) followed by non-Hodgkin lymphoma 5.4% (ASR 2.4), lung cancer 4.0%, (ASR 2.8) and prostate cancer 3.3% (ASR 2.5). The most frequent sites of cancer in females by rank order were cervix uteri 34.0% (ASR 18.9), liver 19.4% (ASR 11.2), breast 9.2% (ASR +5.5) and ovary 3.2% (ASR 1.6). The overall age standardized incidence rates were 61.0 and 55.7 per 100 000 for males and females respectively.

A total of 149 lymphomas were registered during the period. These include 17 cases of Hodgkin's disease and 132 non-Hodgkin lymphomas. Overall, more than 50% of the lymphomas had histological confirmation of the diagnosis, the rest were registered on clinical grounds. 25 cases of Burkitt's lymphoma were included in the register. With the exception of 1 case of unknown age and another in age group 25–34, all were in the age range of 0–14 years and 30% had histological verification of diagnosis.

28 cases of Kaposi's sarcoma were registered. There were 6 cases diagnosed by lymph node biopsy and one with unknown primary site. Apart from these, all were localized in the lower limb or were of the generalized 'epidemic' disseminated type.

Age-specific incidence rates for the leading cancer sites in males and females are shown in Figures 3 and 4, respectively. Liver cancer begins to increase at younger ages in males than in females and unlike males, incidence rates in females reach a maximum after age 40 years. In men, prostate cancer increases sharply after age 50 and is more common in the oldest age groups. The incidence of cervix cancer rises rapidly in young women, to reach a maximum at age 40. Breast cancer increases less dramatically with age, and becomes more common than cervix cancer after age 40 before declining in the oldest age groups.

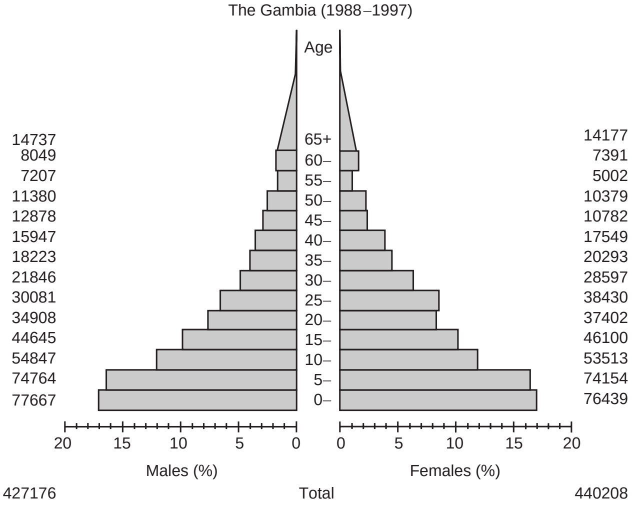

**Figure 2**

 *© 2001 Cancer Research Campaign*

**Table 1** Number of new cancer cases and annual incidence rates by age group. The Gambia, 1988–1997

a. ***Males***

| | Age | | | | | | | | | | | | | |
|---|---|---|---|---|---|---|---|---|---|---|---|---|---|---|
| **Site** | **ICD-10** | **AGE UNK** | **MV*(%)** | **0–14** | **15–24** | **25–34** | **35–44** | **45–54** | **55–64** | **65+** | **All ages** | **% of Total** | **CRUDE RATE** | **ASR** WORLD** |
| Oral Cavity & Pharynx | C00–C13, C14 | 6 | 50 | – | – | 1 | 3 | 3 | 8 | 7 | 28 | 1.7 | 0.5 | 1.3 |
| Oesophagus | C15 | 4 | 12 | – | – | 4 | 1 | 7 | 4 | 6 | 26 | 1.7 | 0.6 | 1.1 |
| Stomach | C16 | 10 | 21 | – | – | 6 | 7 | 15 | 6 | 9 | 53 | 3.5 | 1.2 | 2.3 |
| Colon & Rectum | C18–C21 | 6 | 24 | – | 1 | 2 | 7 | 6 | 6 | 9 | 37 | 2.4 | 0.9 | 1.6 |
| Liver | C22 | 207 | 4 | 8 | 26 | 129 | 157 | 150 | 125 | 93 | 895 | 59.0 | 21.0 | 35.7 |
| Pancreas | C25 | 2 | 0 | – | – | 1 | – | 2 | 6 | 4 | 15 | 1.0 | 0.4 | 0.7 |
| Larynx | C32 | 2 | 19 | – | – | – | 1 | 5 | 5 | 3 | 16 | 1.1 | 0.4 | 0.8 |
| Bronchus & Lung | C34 | 13 | 13 | – | 1 | 3 | 2 | 9 | 15 | 18 | 61 | 4.0 | 1.4 | 2.8 |
| Skin Melanoma | C43 | 1 | 88 | – | – | – | 1 | 3 | 1 | 1 | 8 | 0.5 | 0.2 | 0.3 |
| Skin, other | C44 | 9 | 58 | – | – | 1 | 2 | 3 | 5 | 4 | 24 | 1.6 | 0.6 | 1.1 |
| Kaposi's Sarcoma | C46 | 7 | 61 | 1 | 2 | 1 | 3 | 1 | 1 | 2 | 18 | 1.2 | 0.4 | 0.6 |
| Breast | C50 | 0 | 67 | – | – | – | – | 2 | – | 1 | 3 | 0.2 | 0.1 | 0.1 |
| Penis | C60 | 4 | 33 | – | – | 1 | 5 | 1 | 2 | 2 | 15 | 1.0 | 0.4 | 0.6 |
| Prostate | C61 | 14 | 25 | 1 | – | – | – | 2 | 14 | 20 | 51 | 3.4 | 1.2 | 2.5 |
| Testis | C62 | 1 | 30 | 1 | 2 | 1 | 1 | – | 2 | 2 | 10 | 0.7 | 0.2 | 0.4 |
| Kidney | C64 | 1 | 44 | 10 | 1 | – | 2 | – | – | 2 | 16 | 1.1 | 0.4 | 0.4 |
| Bladder | C67 | 10 | 24 | – | – | – | 2 | – | 3 | 6 | 21 | 1.4 | 0.5 | 1.0 |
| Eye | C69 | 1 | 86 | 5 | 2 | 1 | 2 | 1 | 1 | 1 | 14 | 0.9 | 0.3 | 0.4 |
| Brain & Nervous system | C70–C72 | 0 | 0 | 1 | – | – | 1 | – | – | – | 2 | 0.1 | 0.0 | 0.1 |
| Thyroid | C73 | 2 | | – | – | 1 | 1 | – | 1 | – | 5 | 0.3 | 0.1 | 0.2 |
| Hodgkin's disease | C81 | 3 | 75 | 5 | 2 | – | 1 | – | 1 | – | 12 | 0.8 | 0.3 | 0.3 |
| Burkitt's lymphoma | C83.7 | – | 31 | 13 | – | – | – | – | – | – | 13 | 0.8 | 0.3 | 0.2 |
| Other non-Hodgkin lymphoma | C82–C85, C96 | 15 | 49 | 16 | 4 | 10 | 8 | 6 | 7 | 5 | 71 | 4.6 | 1.6 | 2.2 |
| Leukaemia | C90–C95 | 6 | 54 | 7 | 2 | 2 | 4 | – | – | 1 | 6 | 1.5 | 0.5 | 0.6 |
| Others | O&U | 18 | 40 | 5 | 4 | 5 | 8 | 10 | 9 | 9 | 68 | 4.5 | 1.6 | 2.6 |
| All sites | ALL | 352 | 17 | 75 | 51 | 173 | 222 | 230 | 229 | 208 | 1542 | 101.6 | 36.1 | 61.0 |

© 2001 Cancer Research Campaign b. ***Females***

| | | | | Age | | | | | | | | | | |
|---|---|---|---|---|---|---|---|---|---|---|---|---|---|---|
| **Site** | **ICD-10** | **AGE UNK** | **MV*(%)** | **0–14** | **15–24** | **25–34** | **35–44** | **45–54** | **55–64** | **65+** | **All ages** | **% of Total** | **CRUDE RATE** | **ASR** WORLD** |
| Oral Cavity & Pharynx | C00–C13, C14 | 10 | 42 | 1 | 2 | 1 | 1 | 4 | 3 | 4 | 26 | 1.9 | 0.5 | 1.2 |
| Oesophagus | C15 | 2 | 44 | – | – | 1 | 3 | 1 | – | 2 | 9 | 0.6 | 0.2 | 0.3 |
| Stomach | C16 | 10 | 21 | – | – | 3 | 6 | 3 | 10 | 7 | 39 | 2.8 | 0.9 | 1.9 |
| Colon & Rectum | C18–C21 | 7 | 30 | – | 2 | 6 | 6 | 5 | 4 | 7 | 37 | 2.7 | 0.8 | 1.5 |
| Liver | C22 | 95 | 4 | 2 | 6 | 39 | 36 | 26 | 25 | 35 | 274 | 19.7 | 6.2 | 11.2 |
| Pancreas | C25 | 2 | 23 | – | – | 1 | 2 | 1 | 5 | 2 | 13 | 0.9 | 0.3 | 0.7 |
| Larynx | C32 | 1 | 50 | – | – | – | – | 1 | – | – | 2 | 0.1 | 0.0 | 0.1 |
| Bronchus & Lung | C34 | 7 | 9 | – | 1 | 1 | – | – | – | 2 | 11 | 0.8 | 0.2 | 0.4 |
| Skin Melanoma | C43 | 2 | 62 | – | – | 1 | – | 2 | – | 3 | 8 | 0.6 | 0.2 | 0.4 |
| Skin other | C44 | 9 | 67 | 1 | – | 2 | 2 | 3 | 3 | 1 | 21 | 1.5 | 0.5 | 0.9 |
| Kaposi's Sarcoma | C46 | 2 | 90 | 1 | 2 | 2 | 2 | 1 | – | – | 10 | 0.7 | 0.2 | 0.3 |
| Breast | C50 | 26 | 39 | 2 | 4 | 13 | 26 | 28 | 16 | 15 | 130 | 9.3 | 3.0 | 5.5 |
| Cervix uteri | C53 | 125 | 22 | – | 11 | 78 | 109 | 88 | 41 | 29 | 481 | 34.5 | 10.9 | 18.9 |
| Corpus Uteri | C54–C55 | 20 | 29 | – | 2 | 8 | 12 | 10 | 11 | 9 | 72 | 5.2 | 1.6 | 3.1 |
| Ovary | C56 | 14 | 31 | – | 6 | 6 | 7 | 6 | 2 | 4 | 45 | 3.2 | 1.0 | 1.6 |
| Female genital, other | C51, C52, C58 | 3 | 56 | – | 5 | 1 | 3 | 1 | 1 | 2 | 16 | 1.2 | 0.3 | 0.5 |
| Kidney | C64 | 1 | 33 | 7 | – | – | 1 | 1 | 2 | – | 12 | 0.9 | 0.3 | 0.3 |
| Bladder | C67 | 2 | 13 | – | – | 2 | 1 | 2 | 3 | 5 | 15 | 1.1 | 0.3 | 0.7 |
| Eye | C69 | – | 80 | 9 | 1 | 1 | 2 | 2 | – | – | 15 | 1.1 | 0.3 | 0.3 |
| Brain & Nervous system | C70-C72 | 0 | 100 | – | – | – | – | – | 1 | – | 1 | 0.1 | 0.0 | 0.1 |
| Thyroid | C73 | 3 | 56 | – | – | 2 | – | 3 | 1 | – | 56 | 0.6 | 0.2 | 0.4 |
| Hodgkin's disease | C81 | 1 | 60 | 2 | – | 1 | – | – | – | 1 | 5 | 0.4 | 0.1 | 0.1 |
| Burkitt's lymphoma | C83.7 | – | 31 | 10 | 2 | – | 1 | – | – | – | 13 | 0.9 | 0.3 | 0.2 |
| Other on-Hodgkin lymphoma | C82–C85, C96 | 11 | 48 | 8 | 4 | 3 | 2 | 4 | 1 | 2 | 35 | 2.5 | 0.8 | 1.0 |
| Leukaemia | C90–C95 | 2 | 64 | 4 | 1 | 1 | 3 | 2 | 5 | 2 | 20 | 1.5 | 0.4 | 0.8 |
| Others | O&U | 13 | 17 | 8 | 4 | 8 | 13 | 12 | 6 | 7 | 71 | 5.1 | 1.6 | 2.6 |
| All sites | ALL | 377 | 25 | 60 | 55 | 184 | 239 | 218 | 142 | 140 | 1415 | 101.5 | 32.1 | 55.7 |

*Percentage verified microscopically. **Age standardized to world standard population.

*© 2001 Cancer Research Campaign*

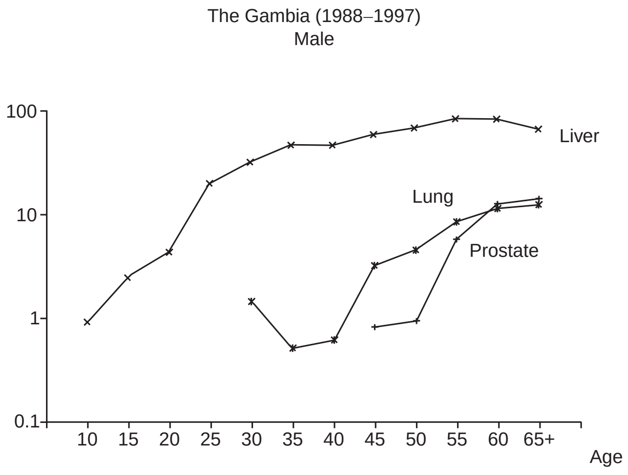

**Figure 3**

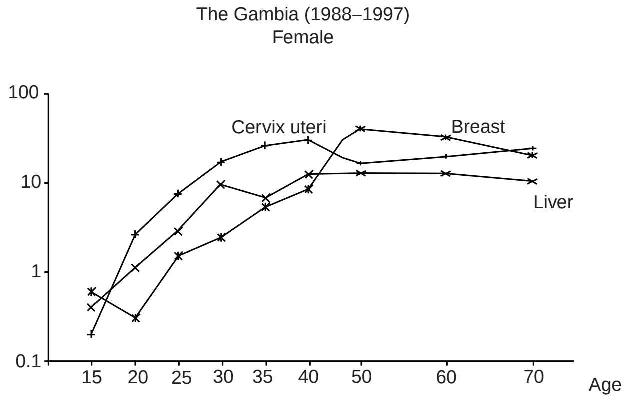

**Figure 4**

### DISCUSSION

There is particular concern with the accuracy of the data when examining the results from cancer registries in the economically underdeveloped countries of Africa. The weak health-care infrastructure and paucity of diagnostic services mean that diagnosis and treatment of cancer, a disease affecting a relatively small number of people and with a very uncertain outcome, has a low priority. The Gambia National Cancer Registry is the only African registry serving an entire national population, the great majority of whom are rural peasant farmers. Nothing is known concerning their behaviour when faced with diseases such as cancer, whether they will present for diagnosis and treatment at local health centres and, having done so, whether they will proceed to tertiary health centres for diagnosis and treatment. Without special studies, the only bases for evaluating completeness of registration are by making comparisons with other similar populations, by studying the stability of incidence rates over time, and by looking at the proportion of cases histologically verified (Parkin et al, 1994).

With respect to stability of rates, there was an increase in the annual numbers of registrations, which had been about 275 per year (and increasing gradually) in 1988–1996, to 480 per year in 1997. This coincided with a modification in registration methods (the permanent out-posting of registry personnel to the major hospitals) and it does suggest that there was some under-registration in the earlier part of the period considered here. As noted in the Methods section, the death certificate registrations are of little use in evaluating completeness, since death certificates are completed only in hospitals (and so do not constitute an independent source of case finding). The very low proportion of cases histologically verified (overall, and for all sites) at least indicates that the registry is not over-dependent on laboratory diagnoses, and that clinical case finding is successful.

Comparisons with other data from West and East Africa (and the USA) are shown in Table 3. Some caution is needed in interpretation, since all of the other African series are from urban centres, with concentrated diagnostic and treatment facilities easily (physically) accessible to the population who are, in any case, probably better informed and more likely to seek medical attention than their rural cousins. Having noted this, it is difficult to know whether the low incidence rates for many sites in The Gambia represent under-diagnosis, under-ascertainment, low incidence rates in a rural population, or genuine geographic variation in risk.

In women, the relative low incidence in cervix cancer (compared to other African registries) suggests that cases are not being diagnosed. There is no reason to suppose that rates of this cancer would be low in rural women (the reverse is the case in India (Jayant et al, 1997; Rajkumar et al, 2000)). Since this is a relatively simple clinical diagnosis (and only 22% of cases that *were* registered had histology), this probably means that many women with cervical cancer do not present to medical attention. Likewise, the very low incidence of prostate cancer in men is quite likely the effect of under-diagnosis rather than any genuine geographic variation in risk.

Even though the recorded incidence of cervix cancer (age standardized rate 18.9 per 100 000) is probably an underestimate, it remains the most common cancer of women. Incidence in young women aged 25–34 (11.6 per 100 000) is relatively high (and, since one quarter of cases have no age recorded, this too is an underestimate). HPV has been unequivocally demonstrated as the major risk factor for cervix cancer (Herrero and Muñoz, 1999), acquisition of the virus being related to multiple sexual partners and early intercourse. The risk is augmented by high parity (Brinton et al, 1989) and possibly by other genital infections (de Sanjose, 1994). Thus, the high incidence rates in West Africa are consistent with early age at marriage, high parity, polygamy and high rates of STD (Meda et al, 1997) in the region. There are no programmes of cervical cancer prevention in the country.

The incidence of liver cancer is probably the most accurately measured. Because of the setting of the GHIS, special attention is paid to identifying all possible cases, and an effort has been made to ensure that diagnostic ultrasound (US) is available in all 3 referral hospitals, and a laboratory service for alpha-fetoprotein (AFP) estimation available to all collaborating hospitals and health centres on simple request. In our data more than 70% of cases have been diagnosed by ultrasound and/or AFP. AFP has been shown to have a sensitivity and specificity for hepatocellular carcinoma of 90% if a cut-off of 400 ng ml$^{-1}$ is used (Kew, 1975). In the Sahelian region, this has been shown to be improved by the addition of ultrasound examination (Tortey et al, 1985). Even so, there has been some fluctuation in the annual numbers of cases registered – including deficits in 1990 and 1994 when the

*© 2001 Cancer Research Campaign*

**Table 2** Percentage of histologically verified (HV) cancer cases

| Site | Number | HV% |
|---|---|---|
| Liver | 1169 | 4 |
| Cervix | 481 | 22 |
| Oral cavity and pharynx | 54 | 46 |
| Oesophagus | 35 | 28 |
| Stomach | 92 | 21 |
| Colon and rectum | 74 | 27 |
| Pancreas | 28 | 23 |
| Bronchus and lung | 72 | 11 |
| Skin melanoma | 16 | 75 |
| Skin, other | 45 | 63 |
| Kaposi's sarcoma | 28 | 76 |
| Breast | 133 | 53 |
| Prostate | 51 | 25 |
| Bladder | 36 | 19 |
| Lymphoma | 149 | 56 |
| Leukaemia | 26 | 59 |
| Others | 468 | 53 |
| All sites | 2957 | 21 |

ultrasound service was out of action – so that the rates even as recorded are something of an underestimate. Nevertheless, they are quite comparable to the contemporary data from Guinea and Mali (Table 3), and from Dakar, Senegal, in the early 1970s (Waterhouse et al, 1982). The rapid increase in incidence with age in young males is typical of Africa (Muñoz et al, 1982).

Epidemiological studies have clearly established chronic carriage of hepatitis B virus (HBV) as a dominant factor in the aetiology of HCC (IARC, 1994). Exposure to aflatoxin is also an important risk factor, particularly in association with chronic carriage of hepatitis B virus (Qian et al, 1994). A high prevalence of HBV infection and its chronic carriage (Whittle et al, 1990) as well as dietary exposure to aflatoxin (Wild et al, 1990) were reported from The Gambia. A case–control study of liver cancer in The Gambia has clearly shown that hepatitis B is the dominant risk factor (Ryder et al, 1992). There is evidence of inter-tribal variation in aflatoxin levels and carriage of hepatitis B surface antigen (HBsAg) in Gambian children, and an association between aflatoxin and chronic carriage of HBsAg has also been observed (Allen et al, 1992). Aflatoxin-albumin adducts levels were found to be higher in the children of the Wollof and Fula tribes (Allen et al, 1992; Wild et al, 1993) than in the other tribal groups. Whether this phenomenon is reflected in a significantly higher rate of liver cancer in adults has not yet been studied. The relatively high incidence of liver cancer, and the presence of a well functioning expanded programme of immunization (EPI) were factors which

**Table 3** Age-Standardized incidence rates: The Gambia, selected African and USA registries a.***Males***

| | West Africa | | | | East Africa | | USA |
|---|---|---|---|---|---|---|---|
| Site | The Gambia 1988–1997 | Mali Bamako[1] 1988–1992 | Guinea Conakry[2] 1992–1995 | Cote d'Ivoire Abidjan[3] 1995–1997 | Zimbabwe Harare[4] 1993–1995 | Uganda Kampala[5] 1995–1997 | US (SEER) Black[1] 1988–1992 |
| Oesophagus | 1.1 | 1.7 | 0.6 | 0.7 | 19.6 | 13.0 | 13.8 |
| Stomach | 2.3 | 19.6 | 6.1 | 3.3 | 12.3 | 7.6 | 14.5 |
| Colon & Rectum | 1.6 | 6.0 | 2.3 | 2.4 | 6.8 | 6.8 | 46.4 |
| Liver | 35.7 | 51.1 | 32.8 | 10.0 | 30.2 | 5.9 | 6.5 |
| Bronchus & Lung | 2.8 | 5.3 | 4.9 | 6.2 | 14.1 | 3.2 | 99.1 |
| Skin Melanoma | 0.3 | 0.5 | 1.3 | 0.7 | 1.5 | 1.1 | 0.7 |
| Prostate | 2.5 | 5.4 | 8.1 | 31.4 | 26.0 | 39.2 | 137.0 |
| Bladder | 1.0 | 10.6 | 3.8 | 2.2 | 8.9 | 2.9 | 11.1 |
| Eye | 0.4 | 1.2 | 0.5 | 0.1 | 1.4 | 3.0 | 0.4 |
| Non-Hodgkin Lymphoma | 2.4 | 2.6 | 2.3 | 3.3 | 4.5 | 7.4 | 12.3 |
| Leukaemia | 0.6 | 0.9 | 0.3 | 1.0 | 2.1 | 1.1 | 9.1 |
| Kaposi's Sarcoma | 0.6 | – | 0.1 | 2.2 | 47.2 | 39.3 | 7.0 |
| All sites | 61.0 | 129.5 | 83.0 | 83.7 | 212.1 | 166.6 | 445.3 |

b. ***Females***

| | | | | | | | |
|---|---|---|---|---|---|---|---|
| Oesophagus | 0.3 | 0.8 | 0.8 | 0.2 | 9.5 | 14.2 | 3.9 |
| Stomach | 1.9 | 10.3 | 5.7 | 4.5 | 11.0 | 5.6 | 5.9 |
| Colon & Rectum | 1.5 | 3.0 | 1.7 | 2.5 | 7.3 | 7.3 | 25.3 |
| Liver | 11.2 | 21.4 | 12.5 | 5.6 | 12.3 | 6.3 | 2.0 |
| Bronchus & Lung | 0.4 | 2.6 | 0.9 | 1.2 | 7.3 | 3.2 | 38.5 |
| Skin Melanoma | 0.4 | 0.9 | 1.1 | 1.4 | 4.4 | 2.2 | 0.5 |
| Breast | 5.5 | 10.2 | 10.9 | 21.4 | 18.6 | 22.0 | 79.3 |
| Cervix uteri | 18.9 | 23.4 | 46.0 | 26.8 | 53.8 | 44.1 | 12.0 |
| Corpus uteri | 3.1 | 0.8 | 1.1 | 2.3 | 4.7 | 4.0 | 11.4 |
| Ovary | 1.6 | 1.0 | 1.8 | 4.0 | 6.1 | 5.3 | 8.1 |
| Eye | 0.3 | 1.0 | 0.3 | 0.9 | 1.9 | 3.0 | 0.3 |
| Thyroid | 0.4 | 1.7 | 0.6 | 1.5 | 3.8 | 5.6 | 3.3 |
| Non-Hodgkin Lymphoma | 1.2 | 0.4 | 1.3 | 2.9 | 3.8 | 5.7 | 7.0 |
| Leukaemia | 0.8 | 2.5 | 0.5 | 2.7 | 3.3 | 1.9 | 5.8 |
| Kaposi's Sarcoma | 0.3 | – | 0.1 | 0.6 | 17.3 | 22.0 | 0.2 |
| All sites | 55.7 | 102 | 110.5 | 98.6 | 210.1 | 179.7 | 272.6 |

[1]Parkin et al, 1997. [2]Koulibaly et al, 2000. [3]Echimane et al, 2000. [4]Chokunonga et al, 2000. [5]Wabinga et al, 2000.

*© 2001 Cancer Research Campaign*

led to the choice of The Gambia for the trial of efficacy of vaccination against hepatitis B (Gambia Hepatitis Study Group, 1987). Recent data have shown the vaccine to be 84% effective against infection and over 90% effective against chronic carriage of HBV (Viviani et al, 1999). A clear effect on incidence of liver cancer should be evident in this study by 2025.

Kaposi's sarcoma remains relatively uncommon, just 18 cases in males and 10 in females. Only 3 of these cases were in elderly men (aged 50 or more); the great majority of the remainder are probably related to HIV. The numbers of cases in young subjects aged under 50 increased from 3 in the 8 years 1988–1995 to 11 in 1996–1997. The incidence remains low, however, in comparison to East Africa (Table 3). Prior to the AIDS epidemic, KS was a relatively rare cancer in West Africa, in comparison with the endemic areas in the east and centre of the continent (Oettlè, 1962). This may have been a reflection of a difference in prevalence of infection with the causative agent human herpes virus 8 (HHV-8) (IARC, 1998), although contemporary seroprevalence of anti-HHV-8 antibodies in Gambia is high, and quite comparable to those areas with high incidence of endemic KS (Lennette et al, 1996; Ariyoshi et al, 1998). The AIDS epidemic arrived rather later in West Africa than in the east and centre of the continent. The current prevalence of infection in The Gambia is 1.1% for HIV-2 and 0.5% for HIV-1 (O'Donovan et al, 2000). It seems that KS is a more frequent complication of HIV-1 infection than HIV-2 – in their study in The Gambia, Ariyoshi et al (1998) found that 8.2% of HIV-1 positive patients with AIDS had KS, compared with 0.8% of AIDS coming years, and already mixed infections with both viruses are reported.

In contrast to an earlier review of the GNCR data (Bah et al, 1990), lung cancer is now among the common male cancers in The Gambia (second in terms of ASR). This phenomenon can be attributed to improved diagnosis, specifically the availability of a bronchoscope at the MRC during recent times. Nevertheless, cancers of the lung, colon and rectum, the most frequent cancers of industrialised countries, remain relatively rare in The Gambia.

### ACKNOWLEDGEMENTS

We wish to thank all health personnel in The Gambia who have contributed to the Registry. Our special thanks go to the doctors, nurses and medical records personnel at RVH, MRC, Bansang hospitals and the major health centres for their continued cooperation and interest in the registry project. We also thank the staff of the WEC mission clinics, Dr S Sisay and staff at the Kololi clinic, Dr S Conteh, Dr T Senghore, and colleagues at the Ahmadiyya Muslim hospital, Drs Palmer, Peters and staff of the Westfield clinic for their continued voluntary notification of cancers diagnosed in their respective health institutions and for allowing us access to their patient records. We acknowledge the diagnostic pathology and serology support provided by Dr FS Oldfield and Mrs Maimuna Mendy during the study period. Mrs Susan Haver of the International Agency for Research on Cancer provided invaluable assistance with the manuscript.

This Registry project was supported through GHIS by the Direzione Generale per la Cooperazione allo Sviluppo of the Ministry of Foreign Affairs of Italy and by the Medical Research Council of Sweden.

### REFERENCES

Allen SJ, Wild CP, Wheeler JG, Riley EM, Montesano R, Bennet S, Whittle HC, Hall AJ and Greenwood BM (1992) Aflatoxin exposure, malaria and hepatitis B infection in rural Gambian children. *Transactions of the Royal Society of Tropical Medicine and Hygiene* **86**: 426–430

Ariyoshi K, Schim Van Der Loeff M, Cook P, Whitby D, Corrah T, Jaffar S, et al. (1998) Kaposi's sarcoma in the Gambia, West Africa is less frequent in human immunodeficiency virus type 2 than in human immunodeficiency virus type 1 infection despite a high prevalence of human herpes virus 8. *J Hum Virol* **1**: 193–199

Bah E, Hall AJ and Inskip HM (1990) The first 2 years of the Gambian National Cancer Registry. *Br J Cancer* **62**: 647–650

Brinton LA, Reeves WC, Brenes MM, Herrero R, De Britton RC, Gaitan E, Tenorio F, Garcia M and Rawls WE (1989) Parity as a risk factor for cervical cancer. *Am J Epidemiol* **130**: 486–496

Chokunonga E, Levy LM, Bassett MT, Mauchaza BG, Thomas DB and Parkin DM (2000) Cancer Incidence in the African Population of Harare, Zimbabwe: Second Results from the Cancer Registry 1993–1995. *Int J Cancer* **85**: 54–59

Cooke A (1998) CANREG-3 Manual. Internal Report No. 98/03. International Agency for Research on Cancer, Lyon, France

De Sanjose S, Muñoz N, Bosch FX, Reimann K, Pedersen NS, Orfila J, Ascunce N, Gonzalez LC, Tafur L, Gili M, et al. (1994) Sexually transmitted agents and cervical neoplasia in Colombia and Spain. *Int J Cancer* **56**: 358–363

Doll R, Payne P and Waterhouse J (1966) Cancer Incidence in Five Continents, Vol. I, Springer-Verlag, Berlin

Echimane AK, Ahnoux AA, Adoubi I, Hien S, M'bra K, D'horpock A, Diomande M, Anongba D, Mensah-Adoh I and Parkin DM (2000) Cancer incidence in Abidjan, Ivory Coast: first results from the cancer registry, 1995–1997. *Cancer*, **89**: 653–63

Herrero R and Muñoz N (1999) Human Papillomavirus and Cancer. In: Newton R, Beral V and Weiss RA (eds) Infections and Human Cancer. *Cancer Surveys* **33**: 75–98. Cold Spring Harbor Laboratory Press

IARC Monographs On The Evaluation Of Carcinogenic Risks To Humans (1994) Volume 59, *Hepatitis viruses*. The International Agency for Research Cancer, Lyon, France

IARC Monographs On The Evaluation Of Carcinogenic Risks To Humans (1998) Volume 70, *Epstein-Barr Virus and Kaposi's Sarcoma Herpesviris/Human Herpes virus 8*. The International Agency for Research Cancer, Lyon, France

Jayant K, Rao RS, Nene BM and Dale PS (1997) In: Parkin DM, Whelan SL, Ferlay J, Raymond L, Young J (eds) Cancer Incidence in Five Continents, Vol VII, IARC Scientific Publications No. 143, International Agency for Research Cancer, Lyon, France

Kew MC (1975) Alpha-fetoprotein. In: *Modern Trends in Gastroenterology, Vol. 5*, Read, A. E. (ed.) p. 91. Butterworth: London and Boston.

Koulibaly M, Kabba IS, Cisse A, Diallo SB, Diallo MB, Keita N, et al. (1997) Cancer incidence in Conakry, Guinea. First Results from the Cancer Registry, 1992–1995. *Int J Cancer* **70**: 39–45

Lennette ET, Blackbourn DJ and Levy JA (1996) Antibodies to human herpes virus type 8 in the general population and Kaposi's sarcoma patients. *Lancet* **348**: 858–861

Meda N, Sangare L, Lankoande S, Sanou PT, Compaore PI, Catraye J, Cartoux M and Soudre RB (1997) Pattern of sexually transmitted diseases among pregnant women in Burkina Faso, West Africa: potential for a clinical management based on simple approaches. *Genitourin Med* **73**: 188–193

Oettlè AG (1962) Geographic and racial differences in the frequency of Kaposi's sarcoma as evidence of environmental or genetic causes. *Acta UICC* **18**: 17–54

O'Donovan D, Ariyoshi K, Milligan P, Ota M, Yamuah L, Sarge-Njie R and Whittle H (2000) Maternal plasma viral RNA levels determine marked differences in mother-to-child transmission rates of HIV-1 and HIV-2 in The Gambia. MRC/Gambia Government/University College London Medical School working group on mother-child transmission of HIV. *AIDS* **14**: 441–448

Parkin DM, Chen VW, Ferlay J, Galceran J, Storm HH and Whelan SL (1994) Comparability and Quality Control in Cancer Registration, IARC Technical Report No. 19, International Agency for Research Cancer, Lyon, France

Parkin DM, Whelan SL, Ferlay J, Raymond L and Young J (eds) (1997) Cancer Incidence in Five Continents, Vol VII, IARC Scientific Publications No. 143, International Agency for Research Cancer, Lyon, France.

Percy C, Van Holten V and Muir C (eds) (1990) International Classification of Diseases for Oncology, Second Edition, World Health Organization, Geneva, Switzerland

Qian GS, Ross RK, Yu MC, Yuan JM, Gao YT, Henderson BE, Wogan GN and Groopman JD (1994) A follow-up study of urinary markers of aflatoxin exposure and liver cancer risk in Shangai, People's Republic of China. *Cancer Epidemiol Biomarkers Prev* **3**: 3–10

Rajkumar R, Sankaranarayanan R, Esmi A, Jayaraman R, Cherian J and Parkin DM (2000) Leads to cancer control based on cancer patterns in a rural population in South India. *Cancer Causes and Control* **11**: 433–439

Ryder WR, Whittle HC, Sanneh ABK, Ajdukiewicz AB, Stevenson T and Yvonnet B (1992) Persistent Hepatitis B Virus Infection and Hepatoma in The Gambia, West Africa. *Am J Epidemiol* **136**: 1122–1131

Smith PG (1992) Comparison between registries: age standardized rates. In: Parkin DM, Whelan SI, Ferlay J, Raymond I and Young J (eds) (1997) Cancer Incidence in Five Continents, Vol VII, IARC Scientific Publications No. 143, International Agency for Research Cancer, Lyon, France, pp. 865–870

The Gambia Hepatitis Study Group: Hall AJ, Inskip HM, Loik F & 14 others (1987) The Gambia Hepatitis Intervention Study. *Cancer Res* **47**: 5782

Tortey E, Coursaget P, Cotty T & 6 others (1985) Real time ultrasonography in the detection of primary liver cancer in intertropical Africa. *Lancet* **i**: 514

Viviani S, Jack A, Hall AJ, Maine N, Mendy M, Montesano R and Whittle HC (1999) Hepatitis B vaccination in infancy in the Gambia: protection against carriage at 9 years of age. *Vaccine* **17**: 2946–2950

Wabinga HR, Parkin DM, Wabwire Mangen F and Nambooze S (2000) Trends in cancer incidence in Kyadondo County, Uganda 1960–1997. *Brit J Cancer* **82**: 1585–1592

Waterhouse JAH, Muir CS, Shanmugaratnam K and Powell J (eds) (1982) Cancer Incidence in Five Continents, Vol IV, IARC Scientific Publications no. 42, p. 210. International Agency for Research on Cancer, Lyon, France

Whittle H, Inskip H, Bradley AK, et al. (1990) The pattern of childhood hepatitis B infection in two Gambian villages. *J Infect Dis* **161**: 1112–1115

Wild CP, Jiang Y-Z, Allen SJ, Jansen LAM, Hall AJ and Montesano R (1990) Aflatoxin-albumin adducts in human sera from different regions of the world. *Carcinogenesis* **11**: 2271–2274

Wild CP, Fortuin M, Francesco D, Whittle HC, Hall AJ, Roland W and Montesano R (1993) Aflatoxin, Liver Enzymes, and Hepatitis B Virus Infection in Gambian Children. *Cancer Epidemiol Biomarkers and Prev* **2**: 1–7

*© 2001 Cancer Research Campaign*
